# Management of delayed perforation after esophageal endoscopic submucosal dissection using a vacuum-assisted closure stent

**DOI:** 10.1055/a-2599-7664

**Published:** 2025-05-28

**Authors:** Francesco Vito Mandarino, Gabriele Altieri, Giuseppe DellʼAnna, Alberto Barchi, Francesco Azzolini, Silvio Danese, Edi Viale

**Affiliations:** 1 9372 Gastroenterology and Gastrointestinal Endoscopy Unit, IRCCS Ospedale San Raffaele, Milan, Italy; 2 18985 Vita-Salute San Raffaele University, Milan, Italy; 3 27288 Gastroenterology and Gastrointestinal Endoscopy Unit, IRCCS San Donato Hospital, San Donato Milanese, Italy

The vacuum-assisted closure stent (VAC-stent) is a novel approach for managing esophageal defects, combining endoscopic vacuum therapy and metal stent support to promote effective tissue healing 1 .

We present a case of esophageal perforation following circumferential endoscopic submucosal dissection (ESD), which was successfully managed with a VAC-stent ( Video 1 ).

Management of delayed perforation following circumferential esophageal endoscopic submucosal dissection using a vacuum-assisted closure stent.Video 1

A 59-year-old man with a 40-mm lesion involving 75% of the esophageal circumference (Paris 0-IIa + Is), arising in Barrett’s esophagus (Prague C4M5) and consistent with adenocarcinoma ( Fig. 1 ), was referred to our unit. Computed tomography scans excluded metastatic disease, and after multidisciplinary consultation, ESD was planned.

**Fig. 1:**
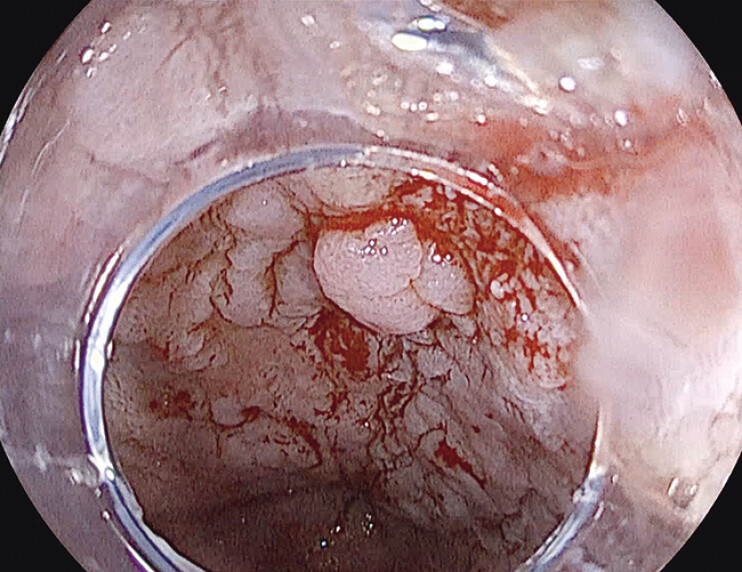
Baseline esophagogastroduodenoscopy with acetic acid chromoendoscopy showing the lesion in the context of Barrett’s esophagus.

Circumferential ESD was performed using a triple-tunnel approach. After the resection, muscular tears were closed with through-the-scope clips ( Fig. 2 ), and pre-emptive endoscopic vacuum therapy was applied to enhance granulation and healing.

**Fig. 2:**
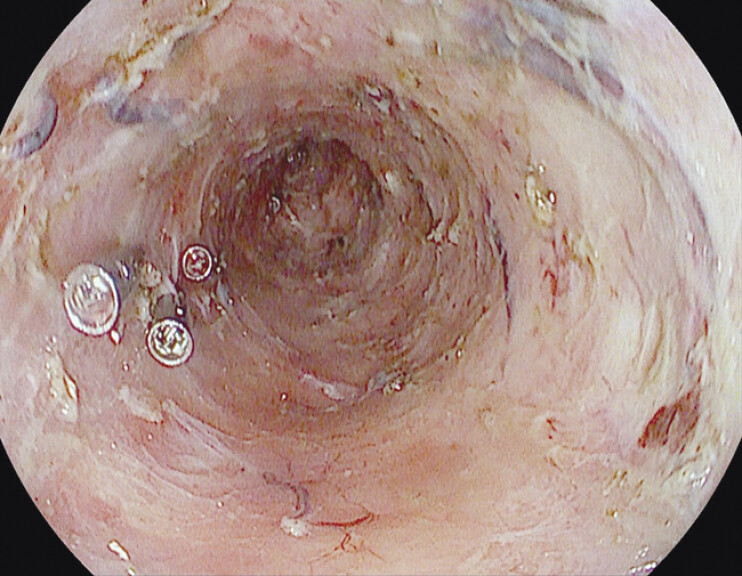
The mucosal defect after endoscopic submucosal dissection. Through-the-scope clips were placed on mild muscular trauma.

Owing to rising inflammatory markers and fever onset, an esophagogastroduodenoscopy (EGD) was performed on postoperative day (POD) 3. The EGD revealed two <5-mm perforations 4 cm apart, and a VAC-stent was placed to bridge the defects. At the follow-up EGD on POD 9, a single defect remained, and a new VAC-stent was placed. The patient showed progressive clinical improvement and successfully resumed a liquid diet. By POD 15, the perforations had healed. The patient was discharged in good clinical condition on POD 18.

At the EGD on POD 30, homogeneous healing tissue was observed, with no stricture detected at the resection site ( Fig. 3 ).

**Fig. 3:**
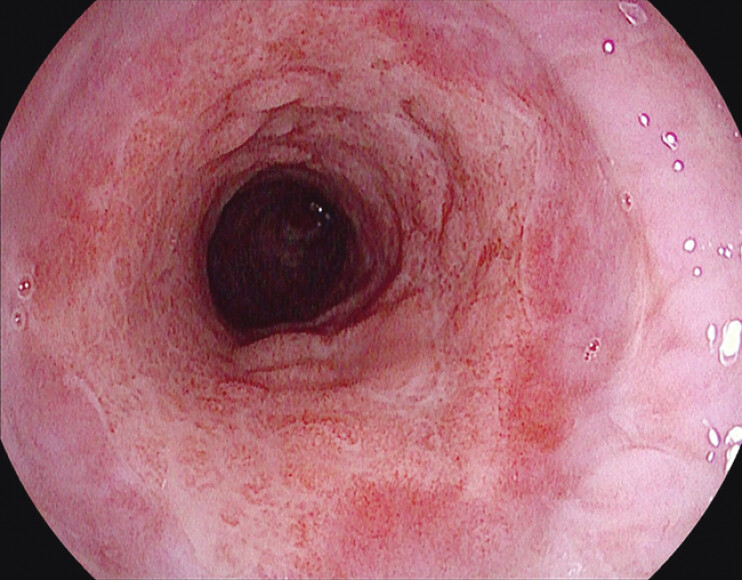
Esophageal defects healed after 30 days of vacuum-assisted closure stent treatment.

Histological analysis of the ESD specimen revealed moderately differentiated adenocarcinoma with submucosal invasion (pT1b [sm1] pNX G2), with R0 resection achieved and no high risk pathological features. An oncological and endoscopic follow-up was planned.

Our case demonstrates that the VAC-stent is a safe and effective method for treating perforations following esophageal ESD. In cases of circumferential ESD, VAC-stent use may also help prevent strictures.

Endoscopy_UCTN_Code_CPL_1AH_2AZ_3AD

## Notes

Endoscopy E-Videos https://eref.thieme.de/e-videos
*E-Videos* is an open access online section of the journal *Endoscopy* , reporting on interesting cases and new techniques in gastroenterological endoscopy. All papers include a high-quality video and are published with a Creative Commons CC-BY license. Endoscopy E-Videos qualify for HINARI discounts and waivers and eligibility is automatically checked during the submission process. We grant 100% waivers to articles whose corresponding authors are based in Group A countries and 50% waivers to those who are based in Group B countries as classified by Research4Life (see: https://www.research4life.org/access/eligibility/ ). This section has its own submission website at https://mc.manuscriptcentral.com/e-videos .
